# Lateral Nasal Wall Respiratory Epithelial Adenomatoid Hamartoma (REAH): A Diagnostic Conundrum

**Published:** 2018-07

**Authors:** Liang-Chye Goh, Ming-Hui Wan, Gopalan Shashi, Shashi Elangkumaran

**Affiliations:** 1 *Department of Otorhinolaryngology, University of Malaya Medical Center, Kuala Lumpur, Malaysia.*; 2 *Department of Otorhinolaryngology, Hospital Tengku Ampuan Rahimah, Klang, Selangor, Malaysia.*

**Keywords:** Electrocautery, Endoscope, Hamartoma, Nasal cavity

## Abstract

**Introduction:**

This study aims to report a rare case of a respiratory epithelial adenomatoid hamartoma (REAH) of the lateral nasal wall that had initially presented as a fungating mass, similar to that of a sinonasal malignancy, and its complete removal from the lateral nasal wall.

**Case Report:**

We report the case of a 58-year-old woman who presented to us with a chief complaint of recurrent right-sided epistaxis and nasal blockage for the past 4 months, which was progressively worsening. Histopathological examination confirmed the presence of a REAH instead of a sinonasal malignancy. The tumor was surgically excised from the lateral nasal wall using electrocautery under endoscopic guidance. The patient was then carefully followed-up after surgery, and the wound was successfully healed 3 months after the initial surgery. There was no evidence of recurrence 6 months after the initial surgery

**Conclusion:**

This case demonstrates the rare presentation of a REAH, which had arisen from the lateral nasal wall. Clinically, it is difficult to distinguish a REAH from a more notorious mass such as a sinonasal malignancy. Therefore, biopsy is mandatory in all cases of lateral nasal mass in order to rule out malignancy before confirming nasal REAH. Fortunately, as seen in this case, a lateral nasal REAH, once diagnosed, can be safely and easily removed from the lateral nasal wall using electrocautery with good surgical outcomes and a low rate of recurrence.

## Introduction

A hamartoma is a benign tumor-like malformation consisting of a disorganized mixture of tissue cells or inborn errors in tissue development that result in an aberrant proliferation of normal tissue components (1). Hamartomas are known to occur throughout the body; however, they are rather uncommon in the nasal cavity and nasopharynx. This condition was termed as respiratory adenomatoid epithelial hamartoma (REAH) by Wenig and Heffner. These tumors originate from the Schneiderian epithelium, whereby glandular elements arise from the epithelium instead of the seromucous glands (2). This case is a rare presentation of REAH originating from the right lateral nasal wall, which usually indicates the presence of an osteoma, inverted papilloma or a sinonasal malignancy; hence, making the clinical diagnosis difficult (3).

## Case Report

**Fig 1:**
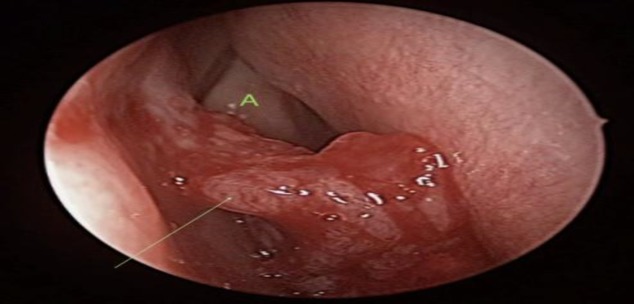
Intraoperative appearance of the mass arising from the mucosa overlying the right lacrimal bone (green arrow) and the adjacent right middle turbinate (green letter A).

A 58-year-old woman with a history of diabetes and hypertension presented to us with chief complaints of recurrent right-sided epistaxis and nasal blockage for the past 4 months, which were progressively worsening. She had no history of headaches, facial numbness, hyposmia, epiphora, aural symptoms, neck swelling or any constitutional symptoms. There was no history of allergy, trauma or smoking in the past. On nasoendoscopic examination, the patient was found to have a mass occupying the right nasal cavity, which was arising from the right lateral nasal wall and free from the nasal septum. The mass was friable and painless on touching (Fig.1). A flexible nasopharyngolaryngoscopic examination of the nasopharynx, oropharynx and hypopharynx and examinations of the oral cavity were unremarkable. A biopsy taken from the mass revealed features suggestive of a REAH.

**Fig 2:**
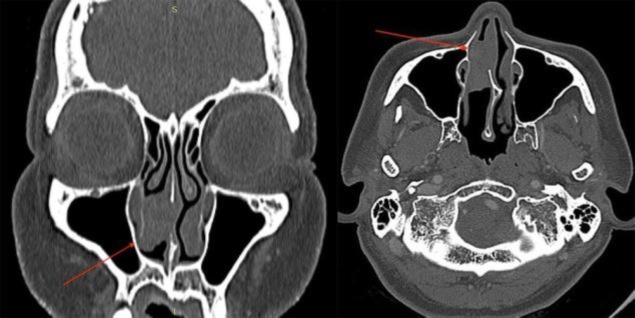
Figure showing the CT paranasal findings of a homogenous non-enhancing mass (red arrow) occupying the right nostril

A contrasted computed tomography (CT) scan of the paranasal sinuses revealed a homogenous non-enhancing soft tissue lesion in the right middle meatus measuring 2 × 2 cm, with no extension into the paranasal sinuses, nasopharynx or contralateral nasal cavity (Fig.2). Upon consultation with the patient, and after obtaining the consent, we proceeded with an endoscopic excision of the right nasal cavity REAH under general anesthesia.

**Fig 3:**
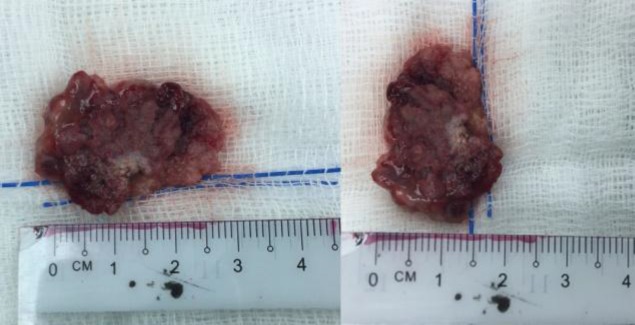
Excised nasal mass measuring approximately 2.5 cm×1.5 cm.

On the basis of these findings, the mass was carefully excised from the right lateral nasal wall using a bipolar electrocautery device. Postoperatively, the patient was discharged with penicillin-group antibiotics and was advised to perform regular nasal douching. An intraoperative biopsy of the 2.5 × 1.5 cm mass (Fig.3) showed polypoidal fragments lined with papilliform projections and its stroma composed of numerous elongated-to-irregular-round glands lined by respiratory-type ciliated columnar epithelium. 

**Fig 4:**
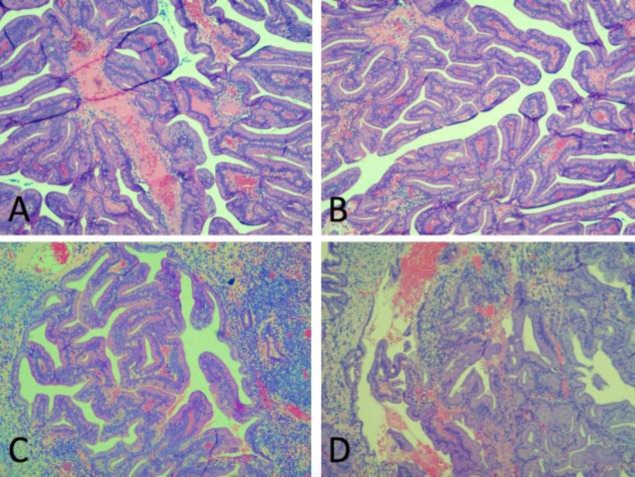
A & B, The polypoidal lesion is lined by papillariform projections (H&E,×10). C & D, The stroma is composed of numerous lobules of elongated,irregular to round glands(H&E,×10and ×4).

**Fig 5:**
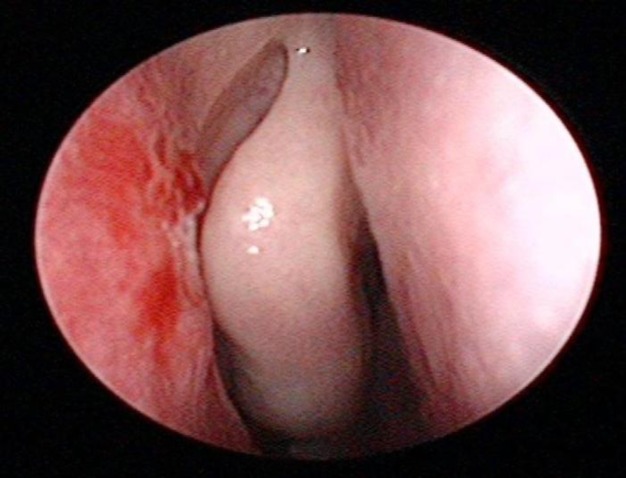
Endoscopic picture taken 2 months after initial surgery showing normal healing tissue and absence of tumor recurrence.

The lesion also exhibited an absence of atypia, necrosis and basement membrane infiltration (Fig.4).In view of the characteristic histopatho-

logical features and benign appearance of the mass endoscopically and radiologically, a diagnosis of REAH was made. The lesion was not confirmed through immunohistochemistry because the characteristic features were obtained through histopathology. The patient was carefully followed-up after surgery and the wound had successfully healed 3 months after the initial surgery. There was no evidence of recurrence 6 months after the initial surgery (Fig.5).

## Discussion

REAH is an uncommon presentation of a nasal mass, which occurs more commonly in men in their third-to-ninth decades of life. There have been approximately 60 cases reported in the literature as case reports or case series and, to a lesser extent, on the immunohistochemical and molecular genetic features of REAH (4). A study conducted by Mühlmeier et al. reported that the incidence of REAH was 1 per 500 of tissue samples obtained from nasal surgeries (5). The condition often arises from the posterior nasal septum and may cause a variety of presenting complaints such as epistaxis, headaches, persistent rhinorrhea and sinusitis, depending on the size and extent of the tumor. REAHs have also been reported to occur in the nasopharynx, paranasal sinuses, lateral nasal wall and the olfactory clefts (4,6). In a case series reported by Hawley et al., the authors categorized nasal REAH as two distinct entities; associated REAH (nasal pathology-associated REAH) and isolated REAH. Associated REAH occurred in about 70% of the studied population, and the remaining cases were isolated REAHs (7). In cases which occurred as an isolated REAH, the tumor had a tendency to arise from the olfactory cleft, but none were reported to have arisen from the mucosa overlying the lacrimal bone, as as seen in the present case. The endoscopic appearance of the mass is often indistinguishable from that of a malignant tumor or an inverted papilloma, as it may be superimposed with infection. This makes it difficult to make an accurate diagnosis. The cornerstone of treatment of a REAH is complete excision of the tumor. As it is a benign tumor, the risk of recurrence or malignant transformation is very low. To the best of the author’s knowledge, there have been no reports of local recurrence of a nasal or nasopharyngeal REAH in the literature (8).

The identification of a nasal REAH remains a diagnostic conundrum as it is often indistinguishable from more common tumors of the nasal cavity, especially squamous cell carcinoma. The distinguishing feature of a REAH is often found during histopathological analysis of the excised tumor. Its histologic pattern is characterized by the abundance of ciliated columnar-lined glandular tissue in a polypoidal fashion. 

Stromal tissues often separate the glands and may be edematous, with evidence of acute and chronic inflammation (9). Although still at its infancy, immunohistochemistry may offer some insight into the differentiation of more notorious lesions found in the nasal cavity. While REAH often shows a preponderance of (Cytokeratin) CK7 positivity, sinonasal carcinoma is more likely to show positivity in CK7, CK20 and CDX-2 panels (9).

Furthermore, Ki-67 panels may serve as a marker of proliferation in REAH, whereby REAH would be expected to have an intermediate labeling index and sinonasal carcinomas are expected to have a high KI-67 labeling index (10). This information could serve as a differentiating feature in REAH and sinonasal carcinoma; however, further studies are needed in order to further confirm this finding.

Histologically, REAH closely resembles findings seen in adenocarcinoma. The World Health Organization has classified malignant sinonasal carcinoma into epithelial intestinal and non-intestinal varieties (11). 

Approximately 20% of low-grade sinonasal non-intestinal-type adenocarcinomas exhibit varied architectural forms with exophytic papillae and glandular patterns (12), with low mitotic figures in low-grade non-intestinal adenocarcinomas. In contrast, high-grade intestinal adenocarcinomas tend to display poorly differentiated features with mitotic activity and pleomorphism. The findings of a low-grade non-intestinal adenocarcinoma can be easily mistaken for a REAH or a seromucinous hamartoma, whereby the latter does not exhibit features of local invasion or a complex growth pattern (13).

Immunochemistry can also be misleading when distinguishing between a REAH and a sinonasal non-intestinal adenocarcinoma, as the latter are also usually consistently positive for CK7 but negative for CK20 and CDX-2 (13). 

Intestinal sinonasal adenocarcinomas, however, tend to show resemblance to mucosa found in normal and neoplastic small intestine and large intestines. These tumors also show variable positivity to CK7, but are usually positive for CK20, CDX-2, villin and MUC2 (13,14), which makes REAH distinguishable from intestinal sinonasal adenocarcinomas.

## Conclusion

When presenting as a lateral nasal mass, a nasal REAH can be safely excised using simple electrocautery with good outcomes. Hence, in patients with a suspected sinonasal malignancy, histological samples should be thoroughly investigated prior to committing to aggressive sinonasal surgeries with resulting severe aesthetic and functional disabilities.

## References

[1] Siti Z, Salina H, Balwant GS (2012). Respiratory Epithelial Adenomatoid hamartoma of the Nasal Septum. Philippine J Otolaryngol Head Neck Surg.

[2] Di Carlo R, Rinaldi R, Ottaviano G, Pastore A (2006). Respiratory epithelial adenomatoid hamartoma of the maxillary sinus: case report. Acta Otorhinolaryngol Ital.

[3] Duzgun Y, Omer S, Berk G, Turan I (2012). Nasal Cavity Masses: Clinico-Radiologic Collaborations, Differential Diagnosis by Special Clues. Open J Med Imag.

[4] Fitzhugh VA, Mirani N (2008). Respiratory Epithelial Adenomatoid Hamartoma: A Review. Head Neck Pathol.

[5] Mühlmeier G, Hausch R, Arndt A, Kraft K, Danz B, Maier H (2014). Respiratory epithelial adenomatoid hamartoma of the nose and nasal sinuses: a rare differential diagnosis of nasal polyposis. HNO.

[6] Lorentz C, Marie B, Vignaud JM, Jankowski R (2012). Respiratory epithelial adenomatoid hamartomas of the olfactory clefts. Eur Arch Otorhinolaryngol.

[7] Hawley KA, Pabon S, Hoschar AP, Sindwani R (2013). The presentation and clinical significance of sinonasal respiratory epithelial adenomatoid hamartoma (REAH). Int Forum Allergy Rhinol.

[8] Himi Y, Yoshizaki T, Sato K, Furukawa M (2002). Respiratory epithelial adenomatoid hamartoma of the maxillary sinus. J Laryngol Otol.

[9] Valerie F, Neena M (2008). Respiratory Epithelial Adenomatoid Hamartoma: A Review. Head Neck Pathol.

[10] Ozolek JA, Barnes EL, Hunt JL (2007). Basal/myoepithelial cells in chronic sinusitis, respiratory epithelial adenomatoid hamartoma, inverted papilloma, and intestinal-type and nonintestinal-type sinonasal adenocarcinoma: an immunohistochemical study. Arch Pathol Lab Med.

[11] Franchi A, Santucci M, Wenig BM, Barnes L, Eveson JW, Reichardt P, Sidransky D (2005). Adenocarcinoma WHO histological classification of tumors of the nasal cavity and paranasal sinuses. Pathology & genetics head and neck tumors.

[12] Jo VY, Mills SE, Cathro HP, Carlson DL, Stelow EB (2009). Low-grade sinonasal adenocarcinomas The association with and distinction from respiratory epithelial adenomatoid hamartomas and other glandular lesions. Am J Surg Pathol.

[13] Leivo I (2016). Sinonasal Adenocarcinoma: Update on Classification, Immunophenotype and Molecular Features. Head and Neck Pathology.

[14] Tilson MP, Gallia GL, Bishop JA (2014). Among sinonasal tumors, CDX-2 immunoexpression is not restricted to intestinal-type adenocarcinomas. Head Neck Pathol.

